# Dual-tuned Coaxial-transmission-line RF coils for Hyperpolarized ^13^C and Deuterium ^2^H Metabolic MRS Imaging at Ultrahigh Fields

**Published:** 2023-08-01

**Authors:** Komlan Payne, Yunkun Zhao, Aditya Ashok Bhosale, Xiaoliang Zhang

**Affiliations:** 1 Department of Biomedical Engineering, State University of New York at Buffalo, Buffalo, NY 14260 USA; 2 Department of Electrical Engineering, State University of New York at Buffalo, Buffalo, NY 14260 USA

**Keywords:** Brain glucose metabolisms, deuterium magnetic resonance spectroscopy, High/low-impedance RF coil, hyperpolarized ^13^C, metabolic imaging, ultra-high field

## Abstract

Information on the metabolism of tissues in both healthy and diseased states holds significant potential for various biomedical applications, including the detection and understanding of tumors, neurodegenerative diseases, diabetes, and other metabolic disorders. Hyperpolarized carbon-13 magnetic resonance imaging (^13^C-HPMRI) and deuterium metabolic imaging (^2^H-DMI) are two emerging X-nuclei used as practical imaging tools to investigate tissue metabolism. However due to their low gyromagnetic ratios (ɣ_13C_ = 10.7 MHz/T; ɣ_2H_ = 6.5 MHz/T) and natural abundance, such method required the use of a sophisticated dual-tuned radiofrequency (RF) coil where the X-nucleus signal is associated with the proton signal used for anatomical reference.

Here, we report a dual-tuned coaxial transmission line (CTL) RF coil agile for metabolite information operating at 7T with independent tuning capability. Analysis based on full-wave simulation has demonstrated how both resonant frequencies can be individually controlled by simply varying the constituent of the design parameters. A broadband tuning range capability is obtained, covering most of the X-nucleus signal, especially the ^13^C and ^2^H spectra at 7T. Numerical results have demonstrated the effectiveness of the magnetic field produced by the proposed dual-tuned ^1^H/^13^C and ^1^H/^2^H CTLs RF coils. Furthermore, in order to validate the feasibility of the proposed design, both dual-tuned CTLs prototypes are designed and fabricated using a semi-flexible RG-405 .086” coaxial cable and bench test results (scattering parameters and magnetic field efficiency/distributions) are successfully obtained.

## I. INTRODUCTION

METABOLIC pathway-based magnetic resonance spectroscopic imaging (MRSI) is a promising tool that can be used to reveal the proper functioning of a biological system [1–7]. This technique provides key information for the diagnosis and treatment monitoring of various diseases, including cancer, diabetes, and neurodegenerative disorders [8–11]. In contrast to MRSI, fluorodeoxyglucose positron emission tomography (FDG-PET), a common metabolic imaging tool, has found success in tumor detection and treatment monitoring in clinical studies by providing high-resolution maps of glucose uptake [12–15]. However, the use of radioactive contrast and the lack of direct information on glucose metabolism have limited its full potential [16, 17]. Conventionally, metabolic MRSI suffers from limited resolution due to its low sensitivity. Recent development demonstrated feasibility of using hyperpolarized carbon-13 magnetic resonance imaging (^13^C- HPMRI) and deuterium metabolic imaging (^2^H-DMI) to probe the tissue metabolic process in vivo, showing much improved sensitivity [18–24]. Unlike FDG-PET, these methods are nonradiative. While DMI method can be performed after oral administration of [6,6’,−^2^H_2_]glucose, ^13^C- HPMRI method involved intravenous injection of hyperpolarized [1^13^C]pyruvate [19, 25]. Both imaging tools, although using different methodology, have proven to be robust and easy to perform in animal brain [26–28] which is further expended to human brain and liver [20, 22, 29–31]. Spectroscopic data acquisition of ^13^C- HPMRI and ^2^H-DMI at 3 Tesla has revealed insufficient inherent spectral resolution [25], leading to a growing interest in high and ultrahigh field studies [26, 32–37] for the proven SNR gain despite technical challenges [38–52]. Due to the low natural abundance and nuclear spin polarization of these X-nuclei (non-hyperpolarized), a weak signal is expected, limiting their signal-to-noise ratio (SNR) and thus quantitative assessment of this metabolic imaging technique [25, 26, 32, 33, 53–55]. Previous studies have indicated that the SNR of the X-nucleus and proton can be significantly enhanced using a dual-tuned RF coil at ultrahigh fields [32, 33, 56–59]. A single coil or two separate coils can both be used to implement a dual-tuned RF coil, owing to their strength and weakness. By adopting the two-coil design, the electromagnetic coupling between the coils can lead to the degradation of the B_1_ field intensity. To address this issue, a decoupling network may be required to reduce the coupling effects between the two coils. However, the inclusion of a decoupling network introduces additional losses in the design, potentially affecting the overall efficiency of the coils. Alternatively, using a quadrature RF coil with a specific geometric configuration can help mitigate the coupling and improve the B1 field intensity. However, this approach can increase the complexity of the coil design [56, 57, 60–63]. In a single-coil dual-tuned design, the coil is intended to be used for multiple resonant frequencies. One of the main challenges with this design is achieving independent tuning between the resonant frequencies. The “two-pole” technique using an LC trap circuit is implemented to design a dual-tuned single-port surface coil with similar B_1_ distribution for both the proton and the hetero nucleus [64]. The same technique is also used for double-tuned birdcage volume coil excited using a single port with double-tuned direct drive or inductive drive [65]. In this work, we proposed a non-array single-coil dual-tuned coaxial transmission line (CTL) RF coil with independent tuning capabilities for X-nuclear MRS imaging aimed at probing metabolite information.

Recently, intensive research has been conducted on CTL RF coils for nuclear magnetic resonance (NMR) at frequencies ranging from high field to ultrahigh field [66–72]. The flexibility of CTL RF coils is to some extent needed to image dynamic anatomy of the human body, as it has lessened the burden of anatomical posture constraints. These types of RF coils are made of coaxial cable with the port connected to the inner conductor and loaded with a gap on the outer shield located opposite to the port [66]. Although they are not truly flexible since lumped components are also integrated within the design, such technology is inexpensive and can be adapted to variations in anatomy. The study of CTL RF coils has revealed multimode operating frequencies associated with the design parameters. While the fundamental frequency is often used to implement receiver RF array coils [66, 68], the second operating mode has a high impedance characteristic and can be used to design a tight-fitting transceiver array [67]. The multimode CTL can be used for a dual-tuned RF coil to study non-proton nuclei (X-nuclei) for magnetic resonance spectroscopy and imaging. By adding gaps in the inner layer or the outer layer, the resonant frequencies of the CTL can be tuned [73]. However, the resonant frequencies of the multimode CTL cannot be tuned independently to the desired frequencies as they are correlated with each other. Recently, independent tuning of both bands can be achieved with two asymmetric gaps on the outer shield conductor of the CTL [74]. The location of the two asymmetric gaps in the outer shield can be carefully selected for the CTL coil through iterative optimization using a full-wave electromagnetic solver to operate at the targeted dual-band frequencies. While this technique allows independent tuning of the two resonant frequencies, in practice, the position of the asymmetric gaps in the outer shield cannot be easily changed to compensate for load variations or cable bending to retune the resonant frequencies. In this work, we propose an alternative dual-tuned CTL RF coil in which the first and second mode resonance frequencies can be tuned independently by simply varying the constituent of the design parameters using lumped components.

In the next section, we elaborate on the development process of our proposed dual-tuned CTL with independent tuning capabilities, namely “shorted end coaxial transmission line” (SECTL). As a proof of concept, we demonstrate that both resonant frequencies can be controlled individually by simply varying the value of the lumped components in the design using full-wave simulation and bench test results. Furthermore, the proposed SECTL design is used to implement dual-tuned ^1^H/^13^C and ^1^H/^2^H RF coils for the ultrahigh field MR applications at 7T, which are valuable for structural and metabolic information detection. Finally, we investigate the efficiency of the magnetic field strength *B*_1,*eff*_ of the proposed dual-tuned SECTL which indicate its feasibility for metabolic clinical application.

## II. Description of Methodology

**Fig. 1.**
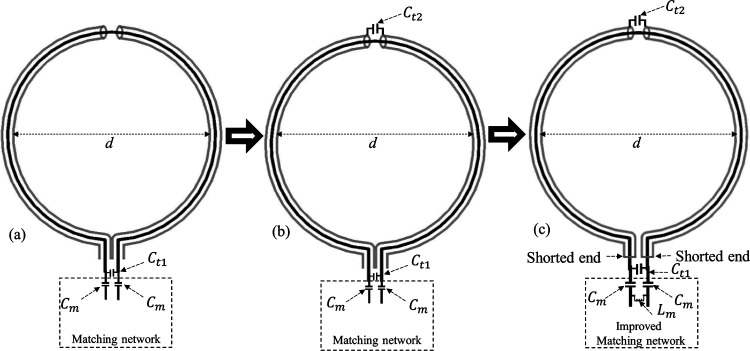
Evolution process of the proposed dual-tuned shorted end CTL coils with independent tuning capabilities. (a) Conventional CTL RF coil for single tuned applications; (b) CTL coil with tuning capacitance (C_t2_) at the gap of the outer shield for single tuned applications; (c) Proposed dual-tuned shorted end CTL with independent tuning capability

The evolution process of the proposed dual-tuned SECTL with independent tuning capabilities is depicted in Fig. 1. The process starts with the conventional CTL RF coil previously introduced [66–68], where C_m_ is the matching capacitor and C_t1_ is used for tuning the resonant frequencies [see Fig. 1(a)]. In such designs, the lumped tuning element C_t1_ simultaneously controls both the first (*f*_*1*_) and second (*f*_*2*_) resonant frequencies, preventing independent tuning capability of the two operating bands. In Fig. 1(b), we introduced another tuning capacitor C_t2_ added to the gap at the outer shield. Simulation analysis has demonstrated that both resonant frequencies (*f*_*1*_ and *f*_*2*_)) are also controlled by the tuning capacitor C_t2_. By isolating the two arms of the outer shielding from each other and shorting the inner conductor to the outer conductor [Fig. 1(c)], we obtained the proposed design namely “shorted end coaxial transmission line” (SECTL). Such alternative design provides independent tuning capability such that the resonant frequencies *f*_*1*_ and *f*_*2*_ are controlled by C_t1_ and C_t2_, respectively. It is worth mentioning that a pi impedance matching circuit is employed to achieve optimal matching at both frequencies. A proper dual-band matching network [65] could be used instead for accurate matching of both resonant frequencies but would require more lumped components which can add resistive losses in the overall design. The proposed SECTL design is used to implement a dual-tuned designed for 7T with independent tuning capabilities for X-nuclear MRS Imaging aiming for metabolite information. To validate the capability of the proposed dual-tuned SECTL with independent control of each resonant frequency, we evaluated the scattering matrix of the design for a fixed value of C_t1_ while varying C_t2_ and vice versa. Furthermore, the simulated/measured *B*_1,*eff*_ map of the dual-tuned SECTL designed for carbon-13 magnetic resonance imaging (^13^C- HPMRI) and deuterium metabolic imaging (^2^H-DMI) is also obtained at their corresponding resonance frequencies.

## III. Proof of the Proposed Dual Tuned Sectl With Independent Tuning Capability

### A. Parametric study results

**Fig. 2.**
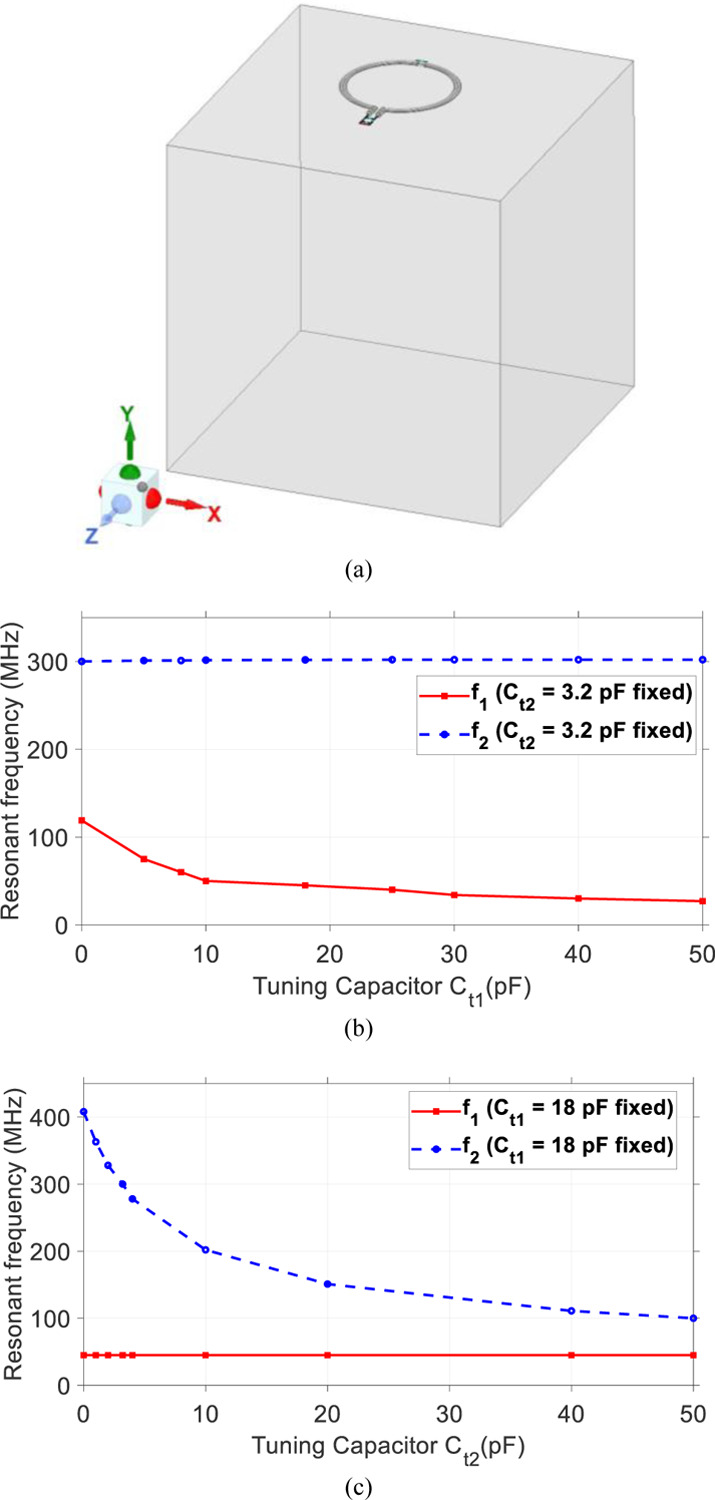
(a) Proposed shorted end coaxial transmission line RF coil loaded with a cuboid phantom. Simulated frequency response result showing the effect of the parametric study. (b) The first resonant frequency *f*_*1*_ is controlled by the tuning capacitor C_t1_; (c) The second resonant frequency *f*_*2*_ is controlled by the tuning capacitor C_t2_. Results show that the two frequencies *f*_*1*_ and *f*_*2*_ can be tuned independently by C_t1_ and C_t2_, respectively.

The proposed SECTL is placed 0.5 cm on top of a tank phantom with dimension 20 × 20 × 20 cm^3^. The conductivity value *σ* = 0.6 S/m and permittivity value *ε*_*r*_ = 0050 are used in the simulation model of the phantom to imitate the human brain tissue properties [see Fig. 2(a)]. The electrical parameters of the material used for the SECTL are the same as the commercial “RG-405 .086” coaxial cable. The diameter of the RF coil is roughly about 8-cm. A full wave simulation using High-Frequency Structure Simulator is used as design analysis for the dual-tuned RF coil. The coil is initially optimized for the second resonant frequency *f*_*2*_ to operate at 300 MHz, the Larmor frequency of the proton ^1^H at 7T (C_t2_ = 3.2 pF). Then, a parametric study is performed on the SECTL by simply varying the value of C_t1_ while other parameters are fixed. The simulations results show that *f*_*1*_ can be independently tuned in a wide range of frequencies (27 MHz – 122 MHz) while *f*_*2*_ remains fixed around 300 MHz [see Fig. 2 (b)]. Note that *f*_*1*_ covers most of the X-nuclear signal (such as ^31^P, ^13^C, ^23^Na, and ^2^H) at 7T. On the other hand, by fixing the value of C_t1_ = 18 Pf (such as *f*_*1*_ operates at 45 MHz, the deuterium spectrum at 7T), while varying the value of C_t2_, the second resonant frequency *f*_*2*_ also shows independent tuning and cover a broad range of spectrum from 100 MHz to 408 MHz [see Fig. 2 (c)].

### B. Design of dual-tuned RF coils

**Fig. 3.**
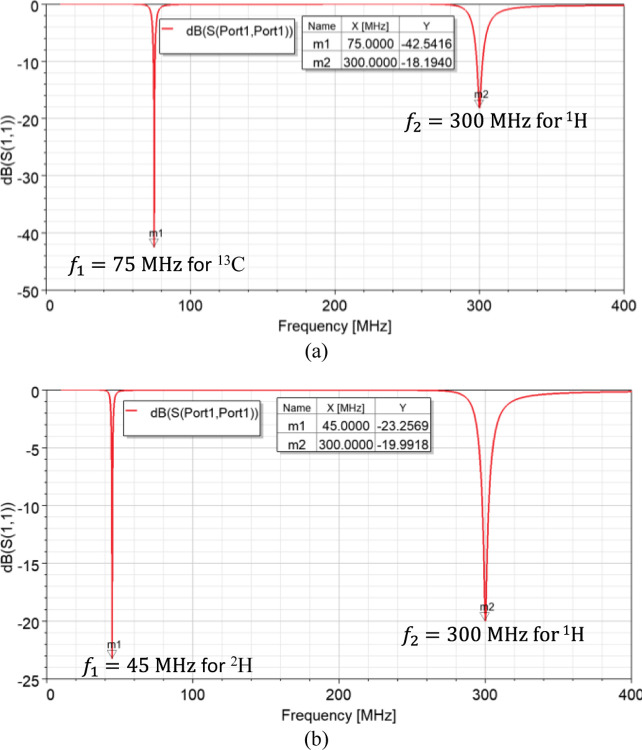
Simulated S-parameter of the dual-tuned SECTL RF coil loaded with the cuboid phantom. (a) Results obtained for the ^1^H/^13^C design where C_t1_ = 6 pF, C_t2_ = 3.2 pF, C_m_ = 22 pF, and L_m_ = 67 nH. (b) Results obtained from the ^1^H/^2^H design where C_t1_ = 18 pF, C_t2_ = 3.2 pF, C_m_ = 16 pF, and L_m_ = 74 nH.

Based on the results obtained from the parametric study, dual-tuned RF coils for metabolites quantification are feasible. Hence, dual-tuned ^1^H/^13^C and ^1^H/^2^H SECTLs RF coils are designed for hyperpolarized carbon-13 MRI and deuterium metabolic imaging (DMI), respectively. Both designs are tuned to their corresponding spectrum and matched to 50 ohms using co-simulation methods. The simulated scattering parameters of the dual-tuned RF coils are shown in Fig. 3. Results indicate a dual-tuned (^1^H/^13^C) response at 7T where the Larmor frequencies of the proton ^1^H and the X-nucleus ^13^C operate at 300 MHz and 75 MHz, respectively. As for the dual-tuned (^1^H/^2^H), the deuterium ^2^H operates at 45 MHz. A good matching is obtained at both frequencies for all the designs using the simple pi-matching network.

### C. Fabrication and Measurement Results

**Fig. 4.**
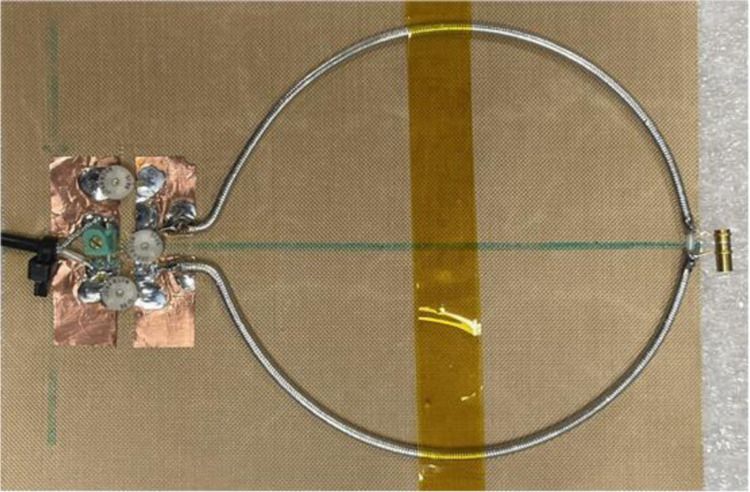
Photograph of the constructed SECTL dual-tuned RF coils with tuning capacitor and inductor. The fabricated RF coil is independently tuned for ^1^H/^13^C or ^1^H/^2^H at 7 Tesla.

**Fig. 5.**
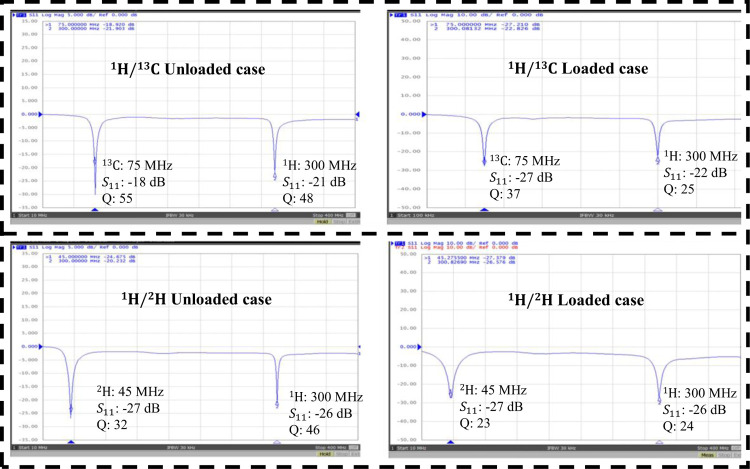
Measured scattering parameters of the fabricated SECTL dual-tuned RF coil for both unloaded and loaded cases. The coil is appropriately tuned at 7T for dual-tuned ^1^H/^13^C operation where the Larmor frequencies of the proton ^1^H and the X-nucleus ^13^C operate around 300 MHz and 75 MHz (a) and for dual-tuned ^1^H/^2^H operation where the Larmor frequencies of the proton ^1^H and the X-nucleus ^2^H operate around 300 MHz and 45 MHz, respectively

A dual-tuned SECTL RF coil adept to be tuned for ^1^H/^13^C or ^1^H/^2^H at 7 Tesla is constructed using the commercial semi-flexible “RG-405 .086” coaxial cable. The inner conductor (with 0.56 mm diameter) and the outer conductor (with 2.2 mm diameter) are isolated using a solid extruded PTFE insulation dielectric material (with 1.7 mm outer diameter, a relative permittivity value of *εr* =2.5 and a loss tangent of tanδ = 0.001). The physical dimensions of the RF coils are the same used in the numerical simulations. Variable capacitors and inductors integrated to the RF coils are soldered at their specific location for matching and tuning purposes. The fabricated dual-tuned RF coil is shown in Fig. 4. A variable trimmer capacitor Johanson 9615 ranging from 5 pF – 25 pF is used for C_t1_ and C_m_ while Johanson 27271 capacitor ranging from 0.6 – 4.5 pF is used for C_t2_. Tunable RF inductor from Coilcraft with inductance value ranging from 65 – 99 nH is used for L_m_. For the loaded case, the fabricated coil is placed on top of a tank water phantom with roughly the same electrical parameter used in the numerical simulation. The scattering parameters of the fabricated RF coil are obtained using a vector network analyzer (from KEYSIGHT, E5061B 100 KHz −3 GHz, Santa Clara, CA, USA). For both unloaded and loaded case scenarios, the fabricated SECTL RF coil is successfully tuned and matched for ^1^H/^13^C or ^1^H/^2^H dual-tuned operation as shown in Fig. 5. The quality (Q) factor is measured from the ratio of the resonant frequency to the −3dB bandwidth for both unloaded (Qunloaded) and loaded (Q-loaded) case. For the ^1^H/^13^C dual channel, the measured Q-ratio (unloaded/loaded) is 1.5 (55/37) for the ^13^C frequency and 1.9 (48/25) for the ^1^H frequency as illustrated in Fig. 5. As for the ^1^H/^2^H dual channel, the measured Q-ratio is 1.4 (32/23) for the deuterium ^2^H frequency and 2 (46/24) for the proton ^1^H frequency. From the measured result, it should be noted that lower Q-ratio is obtained at the X-nucleus (^13^C or ^2^H) compared to the one observed at the proton (^1^H). This situation reflects less coupling between the RF coil and the load at the lower frequency compared to the higher frequency due to the outer shield. For future work, we believe that this issue can be solved by adding more gaps to the outer shield of the RF coil which also will benefit the implementation of dual-tuned array for parallel imaging.

**Fig. 6.**
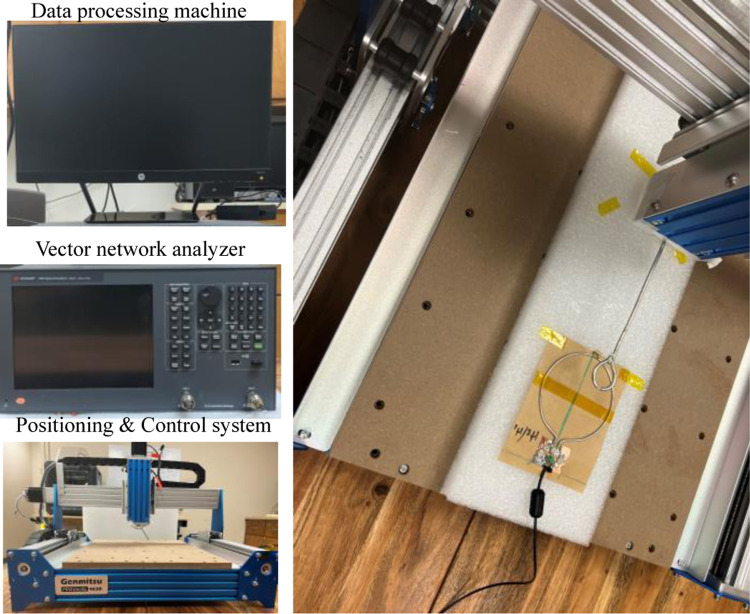
Photograph of the near field measurement setup including the H-field probe, the milling machine, the vector network analyzer, and the data processing machine.

The simulated and measured magnetic field efficiency (*B*_1,*eff*_) generates by the proposed SECTL RF coils are compared at the transversal and coronal planes in a vacuum (unloaded case). The field map is obtained for a 20 × 10 cm^2^ central axial slice and a 10 × 10 cm^2^ coronal slice located 0.5 cm away from the RF coil. The simulated *B*_1,*eff*_ is obtained from the HFSS solver field calculator by normalizing the magnitude of *B*_1_ field to 1 W of accepted power $\left(\left|B\right|/\sqrt{P_{acc}}\right)$ considering the reflected power due to impedance mismatch at the port of the RF coils. As for the measured *B*_1,*eff*_ field, a near field measurement technique similar to the one described in [75] is adopted. As shown in Fig. 6, a sniffer (H-field probe) is used to detect the B_1_ field generated by the dual-tuned RF coil in 3D space. Such sniffer is integrated to a high-resolution router machine (Genmitsu CNC PROVerXL 4030) for accurate control and positioning of the field probe connected to a vector network analyzer (VNA) from Keysight, E5061B, Santa Clara, CA, USA. The raw data obtained from the VNA is processed using MATLAB to compute the measured *B*_1,*eff*_, mapping in different orientations (both in transversal and coronal plane for this experiment) considering cable losses and input impedance matching.

**Fig. 7.**
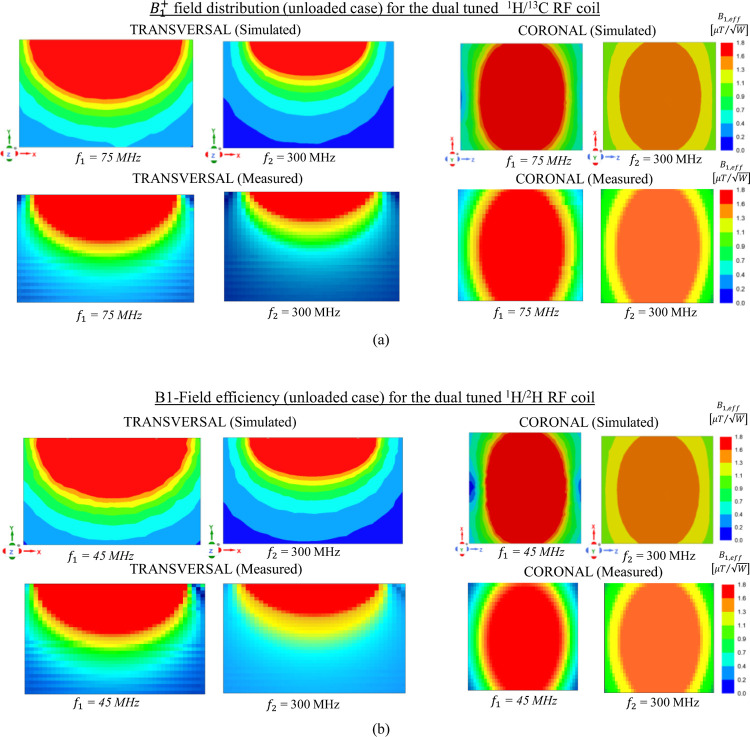
Simulated and measured *B*_1,*eff*_ field distribution (unloaded case) normalized to the accepted input power obtained for both axial and coronal plane; The experimental $B_{+}^{1}$ map is obtained for the 20 × 10 cm^2^ central axial slice and a 10 × 10 cm^2^ coronal slice 0.5 cm away from the RF coil. (a) Field obtained for the dual-tuned ^1^H/^13^C RF coil at 7T; (b) Field obtained for the dual-tuned ^1^H/^2^H RF coil at 7T. Good agreement is obtained between the simulated and measured field maps.

As can be seen in Fig. 7, excellent qualitative and quantitative agreement is obtained between the simulated and the measured $B_{1}^{+}$ mapping in both orientations except for some slight deviation due to the fabrication tolerance and the resolution of the router machine. The dual-tuned ^1^H/^13^C RF coil delivers similar field homogeneity [see Fig. 7(a)] at both frequencies (*f*_*1*_ = 75 MHz, *f*_*2*_ = 300 MHz) which is beneficial for data acquisition of the metabolite information while using the proton for structural imaging and shimming. Likewise, as illustrated in Fig. 7(b), comparable field distribution is also obtained for the dual-tuned ^1^H/^2^H RF coil at its corresponding frequencies *f*_*1*_ = 45 MHz and *f*_*2*_ = 300 MHz. For both RF coils, it can be pointed that, at the lower operating frequency (*f*_*1*_), stronger magnetic flux density is obtained compared to the one obtained at higher frequency (*f*_*2*_). This is due to the increase of radiation losses at higher operating frequency from the coaxial transmission line. Nevertheless, the simulated *B*_1,*eff*,_ field efficiency shown that the dedicated dual-tuned RF coils are feasible and potential candidate for high resolution metabolite images.

## IV. CONCLUSION

We have introduced a dual-tuned coaxial transmission line RF coil with independent tuning capability. The analysis based on full-wave simulation has demonstrated how both resonant frequencies can be controlled individually by simply varying the constituent of the design parameters. A broadband tuning range is obtained, which can cover the proton (high-frequency mode) and most of X-nuclei (low-frequency mode) at 7T. This design characteristic has enabled the implementation of a dual-tuned ^1^H/^13^C for hyperpolarized carbon-13 magnetic resonance imaging (^13^C- HPMRI) and ^1^H/^2^H for deuterium metabolic imaging (^2^H-DMI), both useful for studying tissue metabolism. This modest single-coil design lessens the design complexity of dual-tuned RF coils and, more importantly, as a building block, will be valuable in the design of dual-tuned multichannel RF arrays.
